# Extreme air pollution events in Hokkaido, Japan, traced back to early snowmelt and large-scale wildfires over East Eurasia: Case studies

**DOI:** 10.1038/s41598-018-24335-w

**Published:** 2018-04-25

**Authors:** Teppei J. Yasunari, Kyu-Myong Kim, Arlindo M. da Silva, Masamitsu Hayasaki, Masayuki Akiyama, Naoto Murao

**Affiliations:** 1 Faculty of Engineering, Hokkaido University, Kita-13 Nishi-8, Kita-ku, Sapporo, 060-8628 Japan; 2 Arctic Research Center, Hokkaido University, Kita-21 Nishi-11, Kita-ku, Sapporo, 001-0021 Japan; 3 NASA Goddard Space Flight Center, 8800 Greenbelt Rd., Greenbelt, MD 20771 USA; 4 Japan Automobile Research Institute, 2530 Karima, Tsukuba, 305-0822 Japan; 5 Institute of Environmental Sciences, Hokkaido Research Organization, Kita-19 Nishi-12, Kita-ku, Sapporo, 060-0819 Japan

## Abstract

To identify the unusual climate conditions and their connections to air pollutions in a remote area due to wildfires, we examine three anomalous large-scale wildfires in May 2003, April 2008, and July 2014 over East Eurasia, as well as how products of those wildfires reached an urban city, Sapporo, in the northern part of Japan (Hokkaido), significantly affecting the air quality. NASA’s MERRA-2 (the Modern-Era Retrospective analysis for Research and Applications, Version 2) aerosol re-analysis data closely reproduced the PM_2.5_ variations in Sapporo for the case of smoke arrival in July 2014. Results show that all three cases featured unusually early snowmelt in East Eurasia, accompanied by warmer and drier surface conditions in the months leading to the fires, inducing long-lasting soil dryness and producing climate and environmental conditions conducive to active wildfires. Due to prevailing anomalous synoptic-scale atmospheric motions, smoke from those fires eventually reached a remote area, Hokkaido, and worsened the air quality in Sapporo. In future studies, continuous monitoring of the timing of Eurasian snowmelt and the air quality from the source regions to remote regions, coupled with the analysis of atmospheric and surface conditions, may be essential in more accurately predicting the effects of wildfires on air quality.

## Introduction

On July 25–26, 2014, Hokkaido (the northernmost prefecture in Japan) suffered a serious air pollution and Sapporo city (the most urbanized city in Hokkaido) cautioned its citizens on July 26 for the first time (http://www.city.sapporo.jp/kankyo/taiki_osen/chosa/documents/140912_pm_youin.pdf; hereafter called, Website1) since PM_2.5_ observations were started in Sapporo in 2010 (the information only available in Japanese: http://www.nies.go.jp/igreen/tj_down.html). On July 25, the maximum observed PM_2.5_ in Sapporo was 155 μg m^−3^ (Website 1). The report released by the city of Sapporo on the event suggested that this worsening in air quality was due to smoke transported from Siberian wildfires (Website 1). This study was motivated by this event and aims to better understand the cause of the wildfires, as well as how the smoke reached a remote place, Hokkaido, which significantly affected the air quality in Sapporo.

The recent reports^1,2^ published by the Intergovernmental Panel on Climate Change (IPCC) have attracted much attention, and many people have large concerns regarding the impact of anthropogenic activities on climate change. Today, anthropogenic emissions are higher in India and China^3^, but these emissions will be hopefully reduced in the future, as both countries cut emissions as many developed countries have already done (e.g., Europe^4^, North America^4^, etc.). On the other hand, biomass burning, which includes human-made^5^ and naturally generated (e.g., lightning-induced^6^) wildfires, as well as agricultural waste burnings^7^, also impacts the concentration of particulate matter in the atmosphere^7,8^. Because of developments in technology, satellite remote sensing has been used to detect global distributions of fire hot spots (Fire Counts^9^, FC, with burned area information^10^ or Fire Radiative Power^11,12^, FRP) from various biomass burnings. Those satellite-retrieved data have further been used to develop emission inventories of air pollutants^11–15^ from biomass burnings. Such emissions inventories^11–15^ have been used in modeling the transport of aerosols and their impact on snow as well as in producing re-analysis data with global models^16–21^.

A previous notable study by Westerling *et al*. reported that early snowmelt could generate more wildfires in the following season over the US^22^. Specifically, they found a negative correlation between the center of mass of stream flow, an indicator of the timing of spring snowmelt, and wildfire frequency in western North America, implying that early snowmelt is relevant to wildfire activities^22^. Furthermore, they also reported that warmer conditions in spring and summer with reductions in winter precipitation often happen in years with early snowmelt, during which long-lasting dry season provides more opportunities for active wildfires^22^. This can likely be explained by the early snowmelt induced Wet-First-Dry-Later hydro-climate feedbacks, which was recently suggested by (Lau, W. K. M. *et al*., Submitted, 2018). In fact, drought (i.e., highly dry conditions) is known to be associated with wildfire activities^23,24^, and wildfires can even be predicted using drought information in regions like southern Europe^25^. Based on the these studies (Lau, W. K. M. *et al*., Submitted, 2018)^22–25^, early snowmelt, warm and dry conditions, and wildfire activities are very likely connected with each other. In addition, some modeling studies reported that biomass burning significantly impacts global and regional climates by changing near-surface temperature and cloud properties^26^ and by altering hydro-climate monsoon systems, accelerating snowmelt over the Himalayas and Tibetan Plateau regions due to the mixture of anthropogenic and biomass burning aerosols^27,28^. A global model study recently reported that in the future, wildfires will be more active in the extratropics if global warming exacerbates^29^. Therefore, in the future, it will be more important to monitor the extent of biomass burnings, which can occur via both natural^6^ and human^5,7^ activities. That will be a large concern of general public in terms of air quality and human health around the world.

In this study, we start by focusing on the large-scale wildfires that produced the smoke transported from Siberia to Japan in July 2014, and their significant impact on the air quality in a big urban city in Sapporo, Japan (Website 1). We will also investigate two more similar cases of fires over East Eurasia, both of which produced smoke that reached Hokkaido in Japan and increased levels of PM_2.5_ there^30^. Then, we examine the climatological context in which these three large-scale wildfires with significant impacts on air pollution in a remote place, Hokkaido, could happen. Although our study is based on a limited number of cases, the preliminary knowledge found in this study will give us valuable insight into what we should focus on for future air quality projections and/or its measures and mitigations in regions downwind from the wildfire source regions. More comprehensive study of the relationships among wildfires, surface and atmospheric conditions, and air quality, which is out of focus of this study, would be important for future works. Our outcomes would also provide a basis for future scientific discussion in studying the effect of wildfires on air quality, especially in the region spanning from East Eurasia to Japan.

## Results

### The impact of wildfires on air quality in Sapporo in July 2014 and the pollution events since 2003

**Figure 1:**
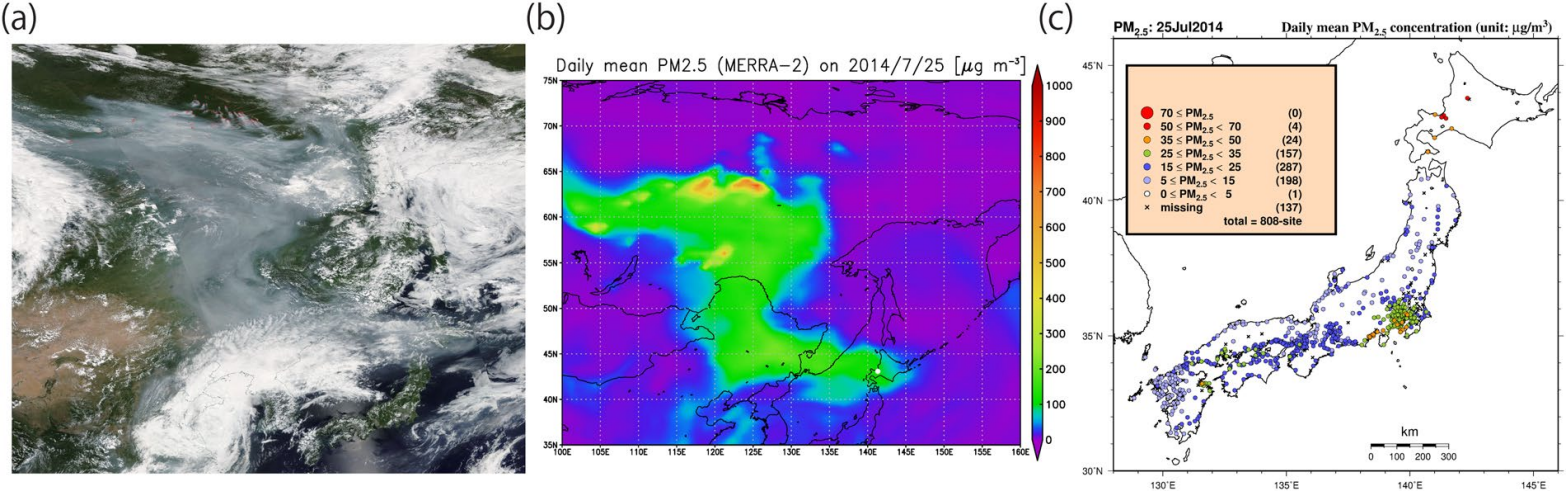
Characteristics of the smoke transport to Hokkaido in Japan and PM_2.5_ distributions on July 25, 2014. (**a**) The Aqua MODIS True Color image with the Fires and Thermal Anomalies (Day and Night) (obtained directly from the NASA Worldview under its “open data policy” with the following permalink (i.e., Google URL Shortener was used to shorten the URL): https://goo.gl/QGfjaj). (**b**) Calculated daily mean PM_2.5_ [μg m^−3^] in Japan Standard Time (JST) with MERRA-2 reanalysis data^20,21,32,48^ and the calculation method of Buchard *et al*. (ref.^17^). The location of Sapporo is shown in white filled circle. (**c**) Daily mean PM_2.5_ [μg m^−3^] on July 25, 2014, from the Japanese observations by the Ministry of the Environment (see Method). Panel (b) was produced with OpenGrADS (http://opengrads.org/; Version 2.1.0.oga.1), which is a sub-project of the main software, Grid Analysis and Display System (GrADS; http://cola.gmu.edu/grads/). Panel (c) was produced with the Generic Mapping Tools (GMT; http://gmt.soest.hawaii.edu), Version 4.5.14.

**Figure 2:**
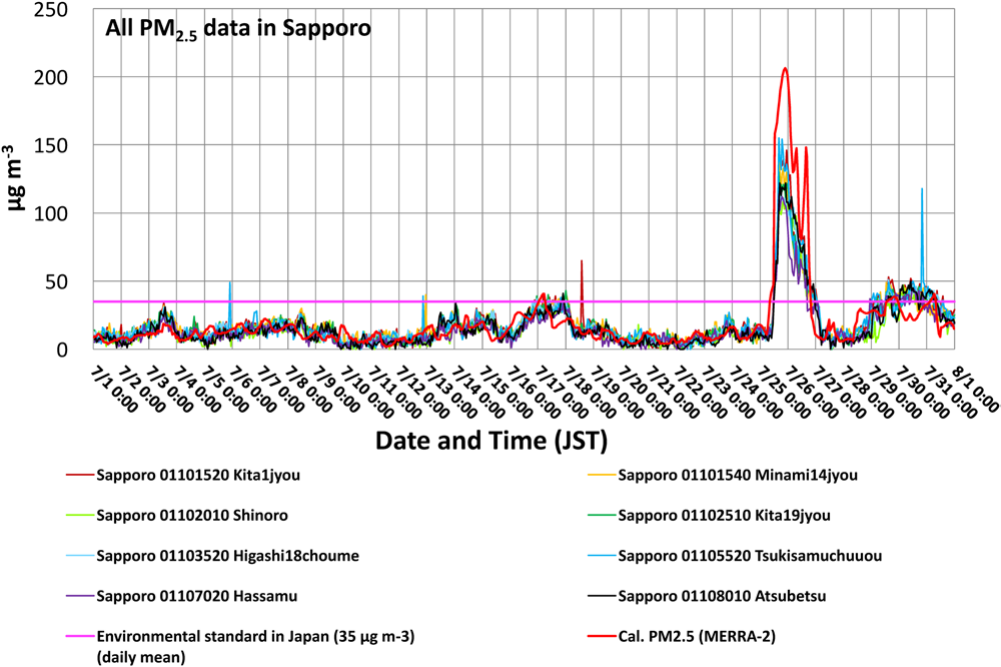
Time series of the observed (eight stations; the validated data) and calculated 1-hourly mean (MERRA-2 with the method of Buchard *et al*.: ref.^17^) PM_2.5_ in Sapporo (Hokkaido, Japan) in July 2014. The solid line in pink is the daily mean environmental standard of PM_2.5_ in Japan (i.e., 35 μg m^−3^; see the URL in the main text).

In July 2014, significantly large-scale wildfires occurred in the Sakha Republic (Russia) and elevated PM_2.5_ levels were observed on July 25, both in the areas directly affected by the fire (i.e., as seen in hot spots) and in faraway locations such as Hokkaido (Japan) (Fig. 1). The observed PM_2.5_ in Sapporo due to the smoke transport peaked on July 25 (Fig. 2; also see Website 1 and ref.^31^), which was closely reproduced by the calculated PM_2.5_ (see the method of ref.^17^) with NASA’s reanalysis data, MERRA-2 (refs^20,21,32^; see Method). Based on the MERRA-2 data (Fig. 1b), in large areas from the Sakha Republic to Hokkaido, the calculated daily mean PM_2.5_ exceeded the daily environmental standard in Japan (35 μg m^−3^, available in Japanese at: http://www.env.go.jp/air/osen/pm/info.html#STANDARD). It is known that this Siberian smoke included much higher amounts of organic carbon (OC) relative to Elemental Carbon (EC) or Black Carbon (BC) (refs^31,33–36^; Supplementary Fig. S1). This is consistent with levels of OC and BC reported in other biomass burning cases from previous studies^7,37^. It is also known that OC and EC has a highly correlated relationship (e.g., the case of agricultural waste burning) (ref.^7^). The MERRA-2 re-analysis data on those carbonaceous aerosol surface mass concentrations in Sapporo well captured the time-varying characteristics of the observed OC and EC increases, although the magnitudes of modeled values were overestimated (Supplementary Fig. S1). These transported carbonaceous aerosols were deposited over Sapporo on July 26, mainly through wet depositions by precipitation rather than through dry deposition and sedimentation processes (Supplementary Fig. S1). The Japan Meteorological Agency (JMA) actually measured the increased precipitation at Sapporo in the afternoon on that day (available in Japanese at: https://goo.gl/2JYNYQ). This July 2014 case, based on our analysis, confirmed that the air quality over larger areas from Eastern Siberia to Northern Japan were significantly affected by highly increased PM_2.5_ due to the Siberian wildfires, which broke out in the Sakha Republic (Fig. 1).

**Figure 3:**
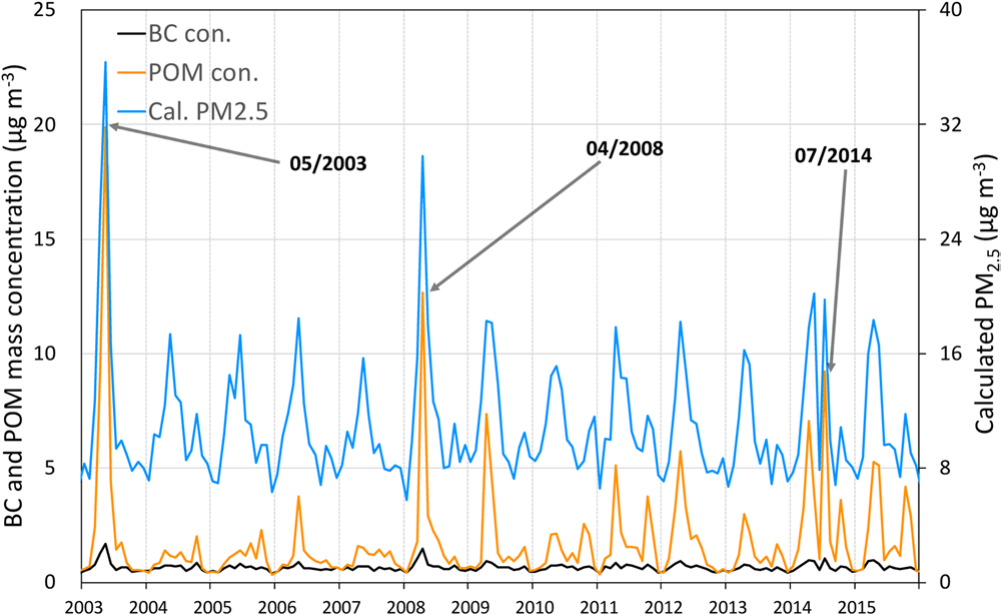
Monthly mean surface BC and POM mass concentrations, and calculated surface PM_2.5_ with the method of Buchard *et al*. (ref.^17^) at Sapporo, Hokkaido, Japan. The top three POM peaks were seen in May 2003, April 2008, and July 2014, respectively.

Then, other questions emerged: What are the main causes of such big wildfires which also significantly impact the air quality in remote places like Hokkaido, Japan? In a previous report^30^, instances of higher levels of PM_2.5_ at Rishiri Island in Hokkaido due to the transport of wildfire smoke were also reported in May 2003 and April 2008. The smoke from the April 2008 case was also transported to Arctic region^38^. Before 2003, the transport of wildfire smoke to Japan was also reported in 1998 and 2002 in a few previous studies^39–41^. Further analysis in this study with the MERRA-2 data at Sapporo also showed that concentrations of Particulate Organic Matter (POM = 1.4 × OC in GEOS-5 model^42^) were significantly increased, and that this, together with increases in BC, contributed to the increases in PM_2.5_ in May 2003 and April 2008, as well as in July 2014 (Fig. 3). We only used the MERRA-2 data from 2003 in this study because of the availability of MODIS fire data from both TERRA and Aqua satellites^20,21^ (see Method). All three cases exhibited highly increased OC, implying that the air quality in Sapporo (Hokkaido)—at least in these three months—was significantly affected by smoke created by wildfires, based on knowledge from previous studies^7,31,33–37^. Although one previous paper^36^ reported aerosol transport including OC from biomass burning from the Siberian region in August 2005, the increase of OC seems to be much smaller compared to these three months above as seen in Fig. 3. Therefore, in the study, we focus on these three months to identify the reasons why these three pollution events happened, and more deeply analyze the climate and environmental conditions over East Eurasia that contributed to the wildfires and the long-range transport of pollutants from the fires. Note that in general, it is very difficult to identify the causes of wildfire ignitions, such as whether they are human-made^5^ or lightning-induced^6^. Therefore, we only identify the characteristics of climate and environmental conditions, which are likely preferable for wildfire ignitions.

### The relationships among snow amounts, climate and environmental conditions, wildfires over Eastern Eurasia, and air pollutions in Hokkaido

**Figure 4:**
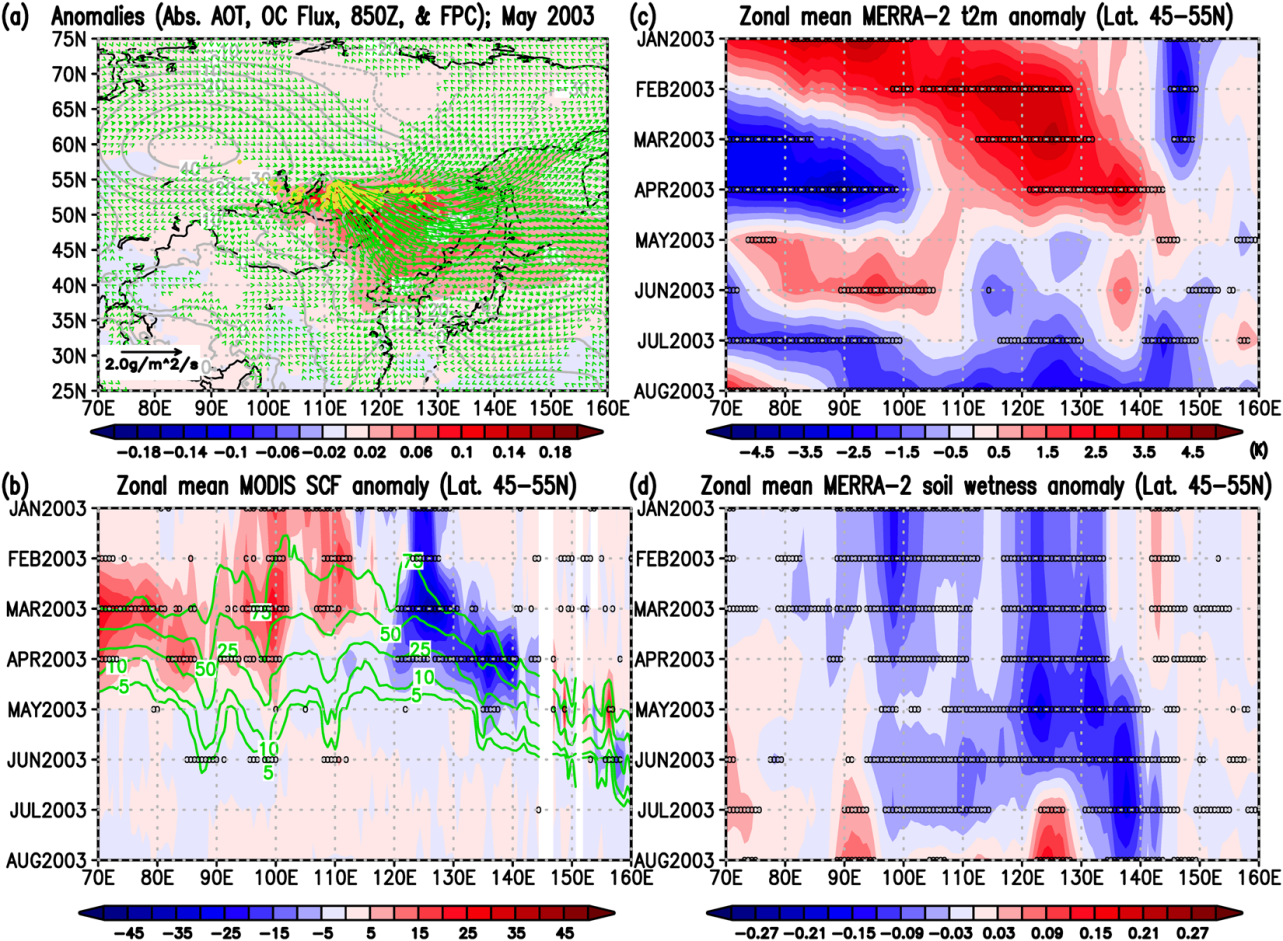
Anomaly relationships among absorbing aerosols, OC (POM) fluxes, fires, snow, and meteorological components for the wildfire case in May 2003. (**a**) Monthly anomalies from the 2003–2015 climatologies on absorbing Aerosol Optical Thickness (AOT) at 550 nm (shaded contour; non unit), Fire Pixel Counts (yellow contour; counts per grid), and geopotential height at 850 hPa (gray contour; m), and longitudinal and latitudinal components of OC (POM) column mass flux (green vector; plotted every two data in longitudes and latitudes if either of the UV components satisfying with the defined unusual condition, see below). (**b**) Zonal mean monthly MODIS Snow Cover Fraction (SCF; %) anomaly (shaded contour) from the 13-year zonal mean monthly climatology (green contour)in the latitudes of 45–55°N. (**c**) Same as (**b**) but for the MERRA-2 2-m surface air temperature anomaly (K). (**d**) Same as (**b**) but for the MERRA-2 surface soil wetness anomaly (non unit). The mark, 0, in black in Panels (b–d) denote that the absolute values of the zonal mean monthly anomaly data from zonal mean monthly climatologies were greater than 3.055*MSE corresponding to the 99% t-based confidence intervals of the climatology (i.e., unusual cases) (see Method). In Panel (b), we further excluded the 0 marks where the monthly zonal mean SCF climatology is smaller than 1% (see Method). Figure 4 was produced with OpenGrADS (http://opengrads.org/; Version 2.1.0.oga.1), which is a sub-project of the main software, GrADS (http://cola.gmu.edu/grads/).

**Figure 5:**
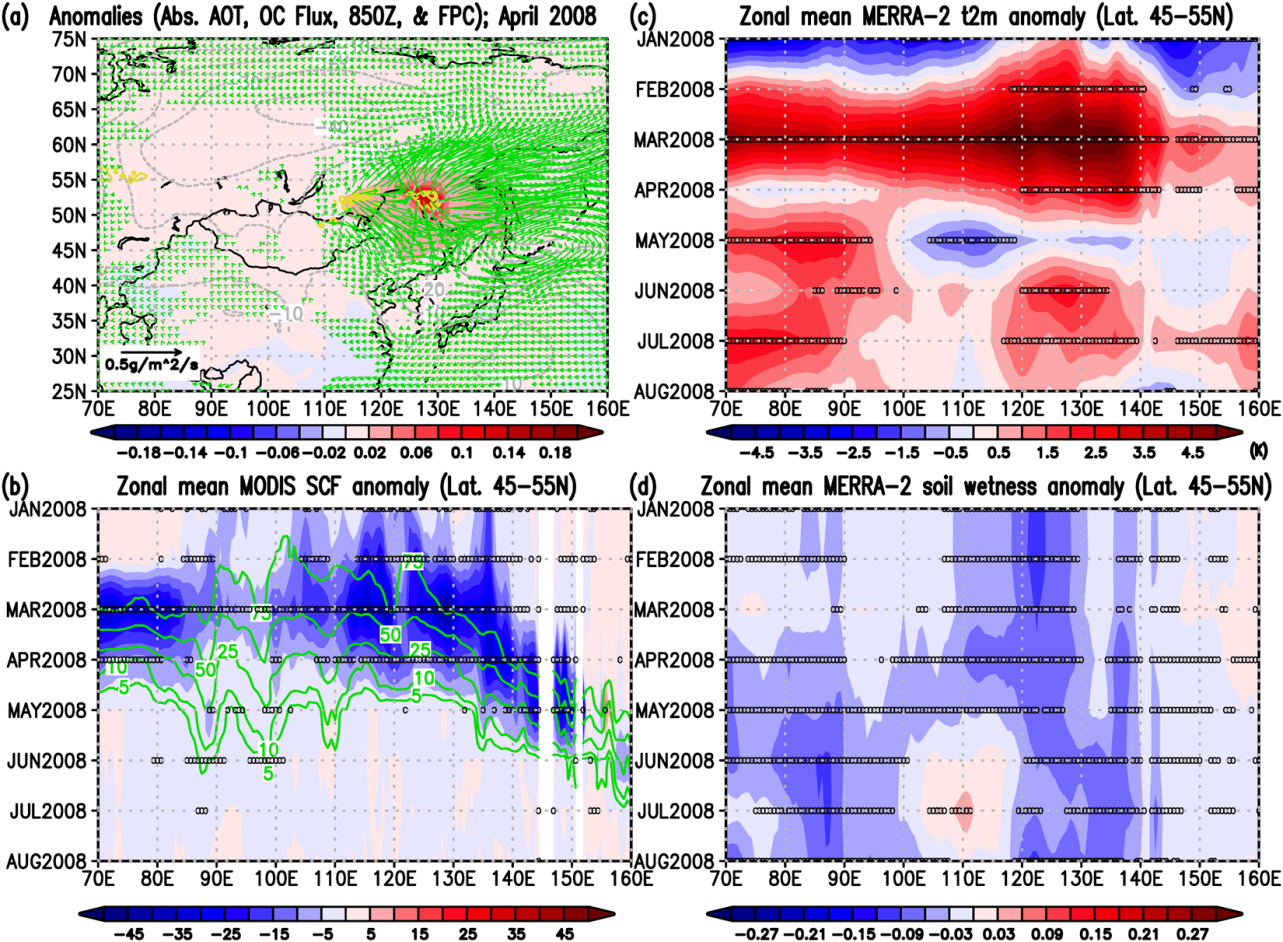
Same as Fig. 4 but for the wildfire case in April 2008. Figure 5 was also produced with OpenGrADS (http://opengrads.org/; Version 2.1.0.oga.1), which is a sub-project of the main software, GrADS (http://cola.gmu.edu/grads/).

**Figure 6:**
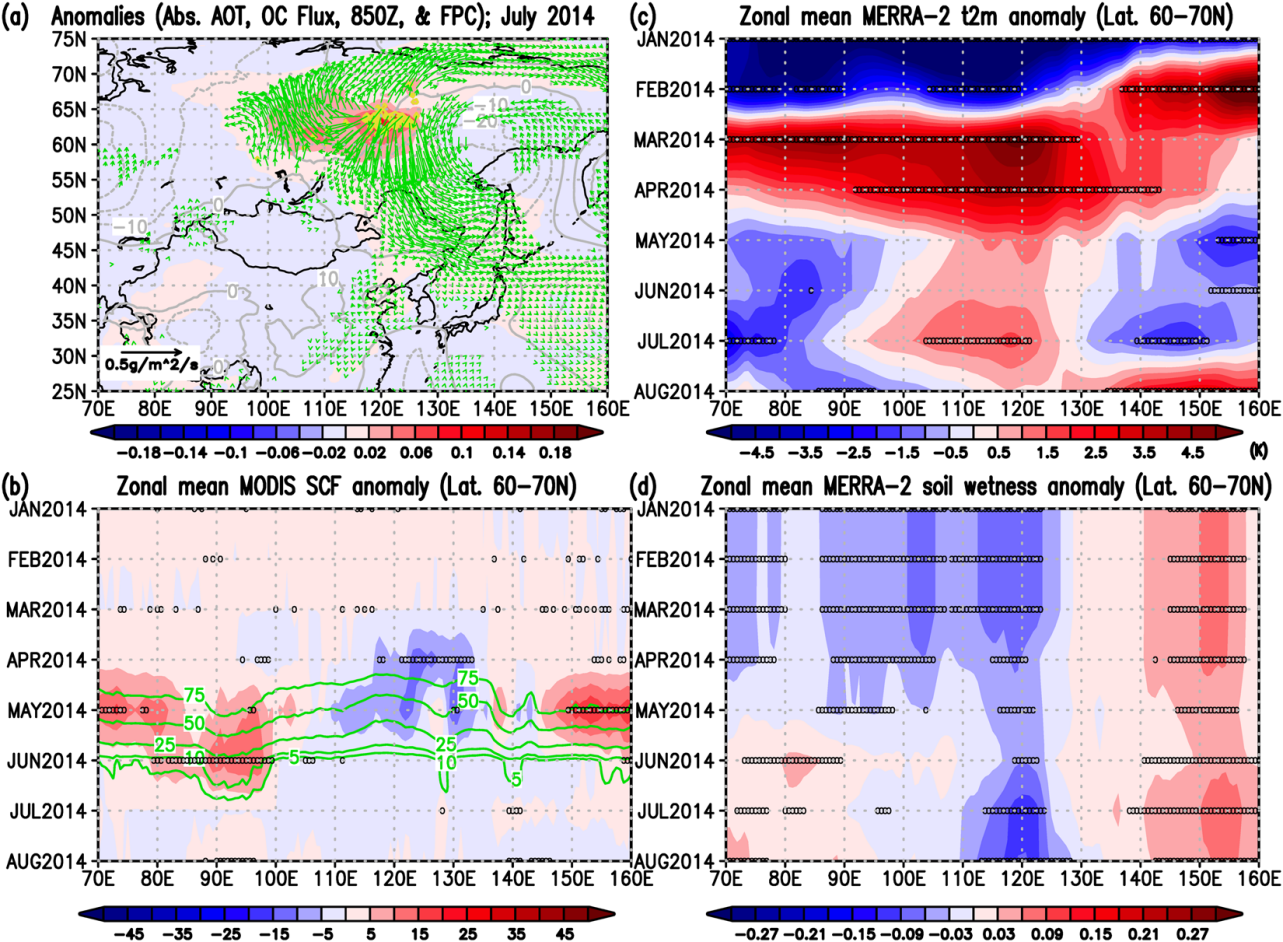
Same as Fig. 4 but for the wildfire case in July 2014. Zonal mean calculations were carried out in the latitudes of 60–70°N for this figure in Panels (b–d). Figure 6 was also produced with OpenGrADS (http://opengrads.org/; Version 2.1.0.oga.1), which is a sub-project of the main software, GrADS (http://cola.gmu.edu/grads/).

In the cases of the May 2003, April 2008, and July 2014 wildfires, we can categorize the spatial smoke characteristics into two patterns. The smoke outbreaks were seen in the eastern parts of Lake Baikal in the latitude zone of 45–55°N due to the May 2003 (ref.^30^) and April 2008 (refs^30,38^) wildfires, and anomalously high pressure systems were dominant over and around Japan (Figs 4, 5, and Supplementary Figs S2 and S3). On the other hand, the wildfires in July 2014 occurred in the Sakha Republic in the latitudes of 60–70°N, a higher latitude than the location of Hokkaido, with a dominant negative geopotential anomaly in the lower troposphere (centered around Amur Oblast) (Fig. 6 and Supplementary Fig. S4). The horizontal OC fluxes in Figs 4–6 clearly showed the smoke transport from the fire-ignition areas all the way to Hokkaido, which is consistent with the locations of positive and negative anomalies of geopotential heights at 850 hPa in Figs S2–S4. However, all three cases share the following common spatiotemporal environmental relationships: (1) unusually small snow cover fractions (SCF) at the location of the large-scale wildfires compared to the SCF climatology (i.e., implications of early snowmelt) (Figs 4b, 5b and 6b) accompanied by significantly warm air temperatures near the surface (Figs 4c, 5c and 6c) in the months preceding the fire; (2) long-lasting unusually low surface soil moisture (i.e., drier conditions) before, during, and after the fires (i.e., from the beginning of the year to the fire month); and (3) worsening of air quality in Sapporo (Hokkaido, Japan) after the fires due to the transport of smoke from the wildfires along synoptic atmospheric circulation motions (Figs 2 and 3). Based on these three cases, these common and clear relationships among early snowmelt, warmer surface conditions, long-lasting drier environmental conditions, trans-boundary particulate matter transport, and worsened air quality in Sapporo gave us important insights for future studies on the connections of climate and air quality due to wildfires over East Eurasia.

## Discussion

Based on some previous studies^30,31,33^ and our analysis, at least three extreme air quality episodes identified in Hokkaido since 2003 were significantly affected by the long-range transport of pollutants from large-scale wildfires in the remote regions of Siberia and East of Lake Baikal. In Fig. 1b, we can see that broad areas suffered from high PM_2.5_ in July 2014. This implies that large-scale wildfires have enormous impacts on the air quality in both local source areas as well as in remote places.

Our findings here indicate that all three large-scale wildfires in Eastern Eurasia were catalyzed by unusually early snowmelts, as seen in Figs 4b, 5b and 6b. As summarized in the introduction, a previous study^22^ over the western United States concluded that an increase in wildfire frequency is associated with warming in the spring and summer, and early snowmelt. In addition, a previous study mention that the fire season in Siberia and Russia started early in 2008 because of unusually low amounts of snow^38^. The results of our three cases on the relationships between early snowmelt and the following wildfires over Eastern Eurasia are consistent with the discussions by those studies^22,38^. Furthermore, in all three cases, unusually early snowmelt over the active wildfire areas, coupled with significant surface warming, likely induced the long-lasting drier conditions in the soil surface (Figs 4–6). These characteristics are also consistent with the known relationship between drought and wildfire activities shown in previous studies^23–25^. Snow amount reductions and surface warming occur simultaneously because snow albedo reductions induce more solar absorption at the surface, as explained in a previous study^19^ and suggested as the Wet-First-Dry-Later mechanism on hydro-climate feedbacks (Lau, W. K. M. *et al*., Submitted, 2018). Although these studies simulated the snow reductions by modeling the snow-darkening effect (Lau, W. K. M. *et al*., Submitted, 2018)^19^, the physical mechanism on the relationship between early snowmelt and surface warming would be the same and can be essentially applied to this study. In our three cases, drier conditions were already seen in January, implying that these three years were unusually dry years. However, the early snowmelt over the fire-ignition areas can somewhat mitigate the dryness for short time periods in the early months because the snowmelt deposit water into the soil. In other words, early snowmelt and stronger surface warming can quickly introduce meltwater to the soil and offset the dry conditions to some extent temporarily, but surface warming can also increase the rate of evaporation from the surface and can eventually return the soil to its unusually dry state. The aforementioned previous studies (Lau, W. K. M. *et al*., Submitted, 2018)^19^ actually showed the increases in evaporation under the snow reduction conditions as a physical mechanism. This could likely maintain the long-lasting drier conditions as shown in this study (Figs 4d, 5d and 6d), which would be explained by the Wet-First-Dry-Later mechanisms on hydro-climate feedbacks (Lau, W. K. M. *et al*., Submitted, 2018). Under these dry conditions, ignitions of wildfires can easily occur and the fires may spread further (i.e., becoming large-scale wildfires) under certain synoptic weather conditions such as blocking high related to Rossby wave breaking, which was reported in the case of Alaskan wildfire^43^. For the 2003 and 2008 cases, smoke from the wildfires likely easily reached Hokkaido because the fires and Hokkaido were located in closer latitudes from West to East (Figs 4 and 5), though high pressure systems also helped transport the smoke to Hokkaido (Figs S2 and S3). However, for the 2014 case, the combination of the fire and pre-fire conditions above and the location of the prevailing negative geopotential anomaly (Figs 6a and S4) were likely essential for the smoke transport to Hokkaido (Fig. 6) because of the long latitudinal distance between the fire areas and Hokkaido.

Early snowmelt in spring is largely affected by albedo reductions, which cause the surface to absorb more solar radiation and accelerate atmospheric heating through a feedback system^44^. In addition, light-absorbing aerosols (LAAs), such as BC and OC have relatively larger contributions to absorptions of solar radiation in a visible band from East Asia to the southern Siberian region compared to LAAs of other regions in the northern hemisphere^19^. A recent study^45^ with a very fine horizontal resolution global model showed that conventional global models in lower horizontal resolutions underestimated the transport of BC to higher latitudes because they failed to accurately model cloud systems around low-pressure systems. This recent study^45^ implies to us that modelled snow-darkening effect caused by BC depositions in higher latitudes would tend to be underestimated in current global models in lower horizontal resolutions. This, of course, will underestimate the simulated snowmelt at higher latitudes in global models in turn. In either case, once early snowmelt enhanced, unusual surface heating should be likely and this further causes the long-lasting drier conditions (Lau, W. K. M. *et al*., Submitted, 2018)^22^. These characteristics were indicated in three cases over East Eurasia in this study. Our conclusions are that all three events of significant air pollution in Sapporo can be traced back to early snowmelt together with surface heating in the fire-ignition areas over East Eurasia under the unusual drier soil conditions starting from the beginning of the years in which the three wildfires occurred. These early snowmelts, along with surface heating in those years, further contributed to the maintenance of long-lasting drier conditions and would likely have provided preferable climate and environmental conditions for active large-scale wildfires in the following months.

In the future, if the modelled projections of snow-darkening effect will be stronger in higher latitudes with improved global models (i.e., those that induce more snowmelts) as implied in a previous study^45^, the frequency of large-scale wildfires, like the July 2014 fire, would likely increase, in addition to the global warming impact on wildfires^29^. Furthermore, if events like the May 2003 and April 2008 fires in the mid-latitudes (i.e., fire outbreaks in the eastern part of Lake Baikal^30^) will become more frequent in the future, the BC and OC aerosols emissions from the wildfires caused by early snowmelts will increase more and that will likely be transported more to higher latitudes in the spring and deposit onto the existing snow as also discussed by a previous study^38^, under some specific atmospheric conditions. Anomalous snow reductions in higher latitudes during spring to later spring (April-May) was clear for the case of 2014 wildfire (Fig. 6b), though the reason for the reduction in snow in this case is out of the scope of this study and will be discussed in future studies. Visible snow albedo can further be reduced and stronger surface heating is possible if the light-absorbing aerosols (LAAs) additionally deposit more onto the snow in higher latitudes, as the fundamental role of the snow-darkening discussed by Yasunari *et al*. (ref.^19^). Such a positive snow-albedo feedback system with snow itself and LAAs on snow can further accelerate snow melting^46^ in addition to ongoing global warming^1,2^.

In this study, we identified the climate, environmental, and air pollution characteristics of three large-scale wildfires from source regions to a remote place. We first started focusing on the transport of Siberian wildfire smoke and its impact on the air quality in Hokkaido in July 2014 (Website 1). We found that monthly variations of POM, PM_2.5_, and BC concentrations from MERRA-2 showed other peaks in May 2003 and April 2004 at Sapporo, which were consistent with the observed PM_2.5_ increases at Rishiri Island in Hokkaido and implied the impact of wildfire smokes^30^. All three cases of the wildfire events had several spatiotemporal characteristics in common, and abnormally low amounts of snow (i.e., snow cover fraction in this study) and dry soil conditions were observed in the locations in the months leading up to and following the fires. Early snowmelts, coupled with stronger surface heating, could somewhat mitigate the dryness temporarily, but the heating effect likely also enhanced evaporation. As a result, these conditions could eventually lead to long-lasting drier conditions because of the Wet-First-Dry-Later hydro-climate feedbacks (Lau, W. K. M. *et al*., Submitted, 2018); that is, the months following unusually low snow conditions can be conducive to wildfires. Eventually, large-scale wildfires happened under these climate and environmental conditions, worsening air quality in remote locations in Hokkaido^30,31,33^ (Website 1; Fig. 2). However, even though all three extreme air quality events investigated here are correlated with earlier snow melt in the regions of the fires, not all wildfires affect air quality in Hokkaido. In addition to the severity of fire associated with dry conditions, synoptic atmospheric circulation conditions are also important, as weather determines the transport and deposition of aerosols. Therefore, starting from this study, we absolutely need more comprehensive studies on these relationships in the future to obtain general relationships between wildfires and climate and environmental conditions.

In the future, the frequency of wildfires has been projected to increase based on global model projections, though the extent of increase in wildfires depends on global warming scenarios^29^. This suggests that we need to continue monitoring changes in climate and environmental conditions relevant to wildfires and in air quality caused by wildfires (i.e., biomass burning), and to develop better monitoring technologies and climate models to accurately project future emissions of smoke (i.e., air pollutions) in advance of international and/or multidisciplinary collaborations with other countries. Over East Eurasia, early snowmelt conditions may be one of many important factors–with combinations of the other climate and environmental factors shown in this study–that likely contributes to wildfires and, ultimately, changes in air quality in regions even far away from the source region. Therefore, in future studies more cases are needed to be analyzed in order to examine more detailed and comprehensive relationships among snow amounts, climate and environmental conditions, fire outbreaks, and the impact of the smoke produced on the air quality in remote places. This study would hopefully be the impetus study for such future studies. Better future projections of large-scale wildfire outbreaks with climate models are essential in order for the people living near wildfire regions and regions downwind to take advance action for more sustainable, healthy lives in those region.

## Methods

In this study, we use the NASA’s state-of-the-art gridded aerosol and meteorological re-analyses data, the Modern-Era Retrospective analysis for Research and Applications, Version 2 (MERRA-2), which was produced by NASA’s Global Modeling and Assimilation Office (GMAO) (ref.^32^), using NASA Goddard Earth Observing System, version 5 (GEOS-5) (ref.^47^). Its horizontal resolution is 0.5° × 0.625° in latitude and longitude^32^. The MERRA-2 includes not only 3D meteorological components but also five aerosol species^20,21,48^ (dust, BC, OC, sulfate, sea salt), using the GOddard Chemistry Aerosol Radiation and Transport (GOCART) Model^42,49–51^ and the following aerosol data assimilation. Both satellite-retrieved and ground-based aerosol optical depth data are assimilated to improve aerosol distribution in MERRA-2^20,21,48^. For the aerosol data assimilation of MERRA-2, the MODIS Aqua and Terra data over both the land and ocean are available for full years starting in 2003, and MODIS Terra and/or AVHRR data only over the ocean were available before 2003 (see Fig. 3 of Randles *et al*., ref.^21^). So in order to use the best aerosol data of MERRA-2, we only use the data from 2003 for our discussion in this study. About more information on the aerosols, aerosol data assimilation method of MERRA-2, and validations of MERRA-2 with aerosol observations, see the relevant papers^20,21,48^. The PM_2.5_ from the MERRA-2 data above were calculated based on the method of Buchard *et al*. (ref.^17^). For the analyses in this study, the absorbing Aerosol Optical Thickness (AOT) at 550 nm was calculated by subtracting the total scattering AOT (variable name: totscatau; non-unit) from the total extinction AOT (variable name: totexttau; non unit). The 2-m air temperature (variable name: t2m; in K), geopotential height at 850 hPa (variable name: h850; in m), and surface soil wetness (variable name: gwettop; non-unit) were also used. The combined monthly MODIS Snow Cover Fraction (SCF; in %) (see at: https://modis.gsfc.nasa.gov/data/dataprod/mod10.php; ref.^52^) retrieved by Terra (MOD10CM) and Aqua (MYD10CM) and the number of fire pixel data (see at: http://feer.gsfc.nasa.gov/) retrieved by the MODIS Terra and Aqua were also used. These MODIS-based data were further re-gridded to the horizontal resolution of the MERRA-2 data (ref.^32^). The main analyses of the MERRA-2 and MODIS data were mainly carried out on the NASA Center for Climate Simulation (NCCS; https://www.nccs.nasa.gov/).

The measured PM_2.5_ (validated data) in Sapporo and Japan were collected and maintained by the National Institute for Environmental Studies (NIES) in the Ministry of Environment (ME) and the daily mean data were calculated in Japan Standard Time (JST). The processes of the observed PM_2.5_ data from provisional data to validated data were reported in the online manual by ME (see its Chapter 6, which is only available in Japanese at: http://www.env.go.jp/air/osen/manual_6th/). The PM_2.5_ data in Sapporo have only been available since 2010 (http://www.nies.go.jp/igreen/tj_down.html). Therefore, comparisons between the observed PM_2.5_ and calculated PM_2.5_ (with the method of ref.^17^) from the MERRA-2 aerosol data^20,21,48^ were only possible in Sapporo for the case of July 2014 in time series. The MODIS True Color Image in Fig. 1a was obtained from the NASA’s Worldview (see at: https://worldview.earthdata.nasa.gov/). The observed EC and OC were measured in Sapporo by the Institute for Environmental Sciences in Sapporo and obtained from the previous study^31^.

For Figs 4–6, we calculated the monthly climatologies for 2003–2015 (13 years) and the anomalies of a variable are defined as deviations from the monthly climatology of the variable for 2003–2015 (13 years). For the statistics, because we have only three cases of the large-scale wildfire events in this study (May 2003, April 2008, and July 2014), it is hard to carry out a t-test for the mean differences. Therefore, we alternatively calculated the corrected sample standard deviations of the monthly climatologies, CSSD (i.e., number of sample, n, minus 1), divided by the square root of number of sample, n, which is the so-called Mean Standard Error (MSE). Then, we used a threshold value of the MSE times 3.055 (i.e., 99% t-based confidence intervals of the data) to judge whether the data at a certain grid points or a certain time were statistically significant or not (i.e., extracting unusual case data). If the absolute value of the anomaly of a variable is greater than MSE*3.055, the data are considered as statistically unusual cases beyond 99% of the t-based confidence intervals of the population mean, i.e., population climatology of the variables (See zero marks in panels b–d in Figs 4–6 and shaded contour areas in Supplementary Figs S2–S4). For the SCF data, we further exclude the zero marks for the monthly zonal mean of SCF anomaly under the MSE*3.055 condition above when values of the monthly zonal mean SCF climatology are smaller than of 1%.

### Data availability statement

The data used in this study are available via contacts to the relevant authors upon requests.

## Electronic supplementary material

Supplementary Information

## References

[1] IPCC in Climate Change. The Physical Science Basis. *Contribution of Working Group I to the Fourth Assessment Report of the Intergovernmental Panel on Climate Change*. (eds. Solomon, S. *et al*.). Cambridge University Press, Cambridge, United Kingdom and New York, NY, USA, pp 996 (2007).

[2] IPCC in Climate Change. The Physical Science Basis. *Contribution of Working Group I to the Fifth Assessment Report of the Intergovernmental Panel on Climate Change* (eds. Stocker, T. F., *et al*.). Cambridge University Press, Cambridge, United Kingdom and New York, NY, USA, pp 1535 (2013).

[3] Ohara T (2007). An Asian emission inventory of anthropogenic emission sources for the period 1980–2020. Atmos. Chem. Phys..

[4] Bond TC (2007). Historical emissions of black and organic carbon aerosol from energy-related combustion, 1850–2000. Global Biogeochem. Cycles.

[5] Syphard AD (2007). Human influence on California fire regimes. Ecol. Appl..

[6] Larjavaara M, Pennanen J, Tuomi TJ (2005). Lightning that ignites forest fires in Finland. Agric. For. Meteorol..

[7] Cheng M-T (2009). Particulate matter characteristics during agricultural waste burning in Taichung City, Taiwan. J. Hazard. Mater..

[8] Wang Q (2007). Impact of biomass burning on urban air quality estimated by organic tracers: Guangzhou and Beijing as cases. Atmos. Environ..

[9] Giglio L, Descloitres. J, Justice CO, Kaufman YJ (2003). An enhanced contextual fire detection algorithm for MODIS. Remote Sens. Environ.

[10] Giglio L, van der Werf GR, Randerson JT, Collatz GJ, Kasibhatla PS (2006). Global estimation of burned area using MODIS active fire observations. Atmos. Chem. Phys..

[11] Ichoku C, Ellison L (2014). L. Global top-down smoke-aerosol emissions estimation using satellite fire radiative power measurements. Atmos. Chem. Phys..

[12] Darmenov, A., & da Silva, A. The Quick Fire Emissions Dataset (QFED): Documentation of versions 2.1, 2.2 and 2.4. NASA/TM–2015–104606, Vol. 38 (available at: https://gmao.gsfc.nasa.gov/pubs/docs/Darmenov796.pdf) (2015).

[13] van der Werf GR, Randerson JT, Collatz GJ, Giglio L (2003). Carbon emissions from fires in tropical and subtropical ecosystems. Glob. Change Biol..

[14] van der Werf GR (2006). Interannual variability in global biomass burning emissions from 1997 to 2004. Atmos. Chem. Phys..

[15] van der Werf GR (2010). Global fire emissions and the contribution of deforestation, savanna, forest, agricultural, and peat fires (1997–2009). Atmos. Chem. Phys..

[16] Tsigaridis K (2014). The AeroCom evaluation and intercomparison of organic aerosol in global models. Atmos. Chem. Phys..

[17] Buchard V (2016). Evaluation of the surface PM_2.5_ in Version 1 of the NASA MERRA Aerosol Reanalysis over the United States. Atmos. Environ..

[18] Yasunari TJ (2014). The GOddard SnoW Impurity Module (GOSWIM) for the NASA GEOS-5 Earth System Model: Preliminary comparisons with observations in Sapporo, Japan. SOLA.

[19] Yasunari TJ, Koster RD, Lau WKM, Kim K-M (2015). Impact of snow darkening via dust, black carbon, and organic carbon on boreal spring climate in the Earth system. J. Geophys. Res. Atmos..

[20] Randles, C. A., *et al*. The MERRA-2 Aerosol Assimilation. *NASA Technical Report Series on Global Modeling and Data Assimilation*, NASA/TM-2016-104606, **45**, pp 143 (available at: https://gmao.gsfc.nasa.gov/pubs/docs/Randles887.pdf) (2016).

[21] Randles CA (2017). The MERRA-2 Aerosol Reanalysis, 1980 onward. Part I: System description and data assimilation evaluation. J. Clim..

[22] Westerling AL, Hidalgo HG, Cayan DR, Swetnam TW (2006). Warming and earlier spring increase Western U.S. forest wildfire activity. Science.

[23] Xiao J, Zhuang Q (2007). Drought effects on large fire activity in Canadian and Alaskan forests. Environ. Res. Lett..

[24] Pausas, J. G. & Fernández-Muñoz, S. Fire regime changes in the Western Mediterranean Basin: from fuel-limited to drought-driven fire regime. *Clim*. *Change*, **110**, 10.1007/s10584-011-0060-6 (2012).

[25] Gudmundsson L, Rego FC, Rocha M, Seneviratne SI (2014). Predicting above normal wildfire activity in southern Europe as a function of meteorological drought. Environ. Res. Lett..

[26] Jacobson MZ (2014). Effects of biomass burning on climate, accounting for heat and moisture fluxes, black and brown carbon, and cloud absorption effects. J. Geophys. Res. Atmos..

[27] Lau KM, Kim MK, Kim KM (2006). Asian summer monsoon anomalies induced by aerosol direct forcing: the role of the Tibetan Plateau. Clim. Dyn..

[28] Lau, W. K. M., Kim, M.-K., Kim, K.-M., & Lee, W.-S. Enhanced surface warming and accelerated snow melt in the Himalayas and Tibetan Plateau induced by absorbing aerosols. *Environ*. *Res*. *Lett*., 5(2), 10.1088/1748-9326/5/2/025204 (2010).

[29] Veira A, Lasslop G, Kloster S (2016). Wildfires in a warmer climate: Emission fluxes, emission heights, and black carbon concentrations in 2090–2099. J. Geophys. Res. Atmos..

[30] Ikeda K, Tanimoto H (2015). Exceedances of air quality standard level of PM_2.5_ in Japan caused by Siberian wildfires. Environ. Res. Lett..

[31] Akiyama, M., Otsuka, H., Akutagawa, T. & Suzuki, H. High-concentration event ofPM_2.5_ in Hokkaido (translated from the Japanese title). *Proceedings of the 21st Hokkaido and Tohoku Branch Meeting of Japan Society for Atmospheric Environment*, Abstract No. 15, pp 2 (in Japanese) (2014).

[32] Bosilovich, M. G., *et al*. MERRA-2: Initial Evaluation of the Climate. *NASA/TM–2015–104606*, 43, pp 139 (available at: https://gmao.gsfc.nasa.gov/pubs/docs/Bosilovich803.pdf) (2015).

[33] Noguchi, I., *et al*. A correlation between black carbon and potassium ion in aerosol -Effect of biomass burning-, *Proceedings of the 53rd Annual meeting of the Japan Society for Atmospheric Environment*, 297 (2012).

[34] Jeong JI, Park RJ, Youn D (2008). Effects of Siberian forest fires on air quality in East Asia during May 2003 and its climate implication. Atmos. Environ..

[35] Yasunari, T. J., *et al*. Atmospheric black carbon and its role. *Saihyou*, 62, 3–42 (Available in Japanese with the English title at: http://www.metsoc-hokkaido.jp/saihyo/pdf/saihyo62/2016_02.pdf) (2016).

[36] Agarwal S, Aggarwal SG, Okuzawa K, Kawamura K (2010). Size distributions of dicarboxylic acids, ketoacids, α-dicarbonyls, sugars, WSOC, OC, EC and inorganic ions in atmospheric particles over Northern Japan: implication for long-range transport of Siberian biomass burning and East Asian polluted aerosols. Atmos. Chem. Phys..

[37] Andreae MO, Merlet P (2001). Emission of trace gases and aerosols from biomass burning. Global Biogeochem. Cycles.

[38] Warneke C (2009). Biomass burning in Siberia and Kazakhstan as an important source for haze over the Alaskan Arctic in April 2008. Geophys. Res. Lett..

[39] Tanimoto H, Kajii Y, Hirokawa J, Akimoto H, Minko NP (2000). The atmospheric impact of boreal forest fires in far eastern Siberia on the seasonal variation of carbon monoxide: Observations at Rishiri, A northern remote island in Japan. Geophys. Res. Lett..

[40] Kato S (2002). The influence of Siberian forest fires on carbon monoxide concentrations at Happo, Japan. Atmos. Environ..

[41] Nagahama Y, Suzuki K (2007). The influence of forest fires on CO, HCN, C_2_H_6_, and C_2_H_2_ over northern Japan measured by infrared solar spectroscopy. Atmos. Environ..

[42] Colarco P, da Silva A, Chin M, Diehl T (2010). Online simulations of global aerosol distributions in the NASA GEOS-4 model and comparisons to satellite and ground-based aerosol optical depth. J. Geophys. Res..

[43] Hayasaka H, Tanaka HL, Bieniek PA (2016). Synoptic-scale fire weather conditions in Alaska. Polar Sci..

[44] Chapin (2005). Role of land-surface changes in Arctic summer warming. Science.

[45] Sato Y (2016). Unrealistically pristine air in the Arctic produced by current global scale models. Sci. Rep..

[46] Aoki T, Tanaka TY (2008). Effect of the atmospheric aerosol depositions on snow albedo (translated from the Japanese title). Tenki.

[47] Rienecker, M. M., *et al*. The GEOS-5 Data Assimilation System - Documentation of Versions 5.0.1, 5.1.0, and 5.2.0. *Technical Report Series on Global Modeling and Data Assimilation*, 27, NASA/TM–2008–104606, pp 118 (available at: http://gmao.gsfc.nasa.gov/pubs/docs/Rienecker369.pdf) (2008).

[48] Buchard V (2017). The MERRA-2 Aerosol Reanalysis, 1980 onward. Part II: Evaluation and case studies. J. Clim..

[49] Chin M, Rood RB, Lin S-J, Müller JF, Thompson AM (2000). Atmospheric sulfur cycle in the global model GOCART: Model description and global properties. J. Geophys. Res..

[50] Chin M (2002). Tropospheric aerosol optical thickness from the GOCART model and comparisons with satellite and sun photometer measurements. J. Atmos. Sci..

[51] Ginoux P (2001). Sources and distributions of dust aerosols simulated with the GOCART model. J. Geophys. Res..

[52] Hall, D. K., Riggs, G. A. & Salomonson, V. V. MODIS/Terra Snow Cover 5-Min L2 Swath 500 m. Version 5. Boulder, Colorado USA: NASA National Snow and Ice Data Center Distributed Active Archive Center (See at: http://dx.doi.org/10.5067/ACYTYZB9BEOS) (2006).

