# Supplementary material for: Extreme air pollution events in Hokkaido, Japan, traced back to early snowmelt and large-scale wildfires over East Eurasia: Case studies
Source: Sci Rep. 2018 Apr 25;8:6413. doi: 10.1038/s41598-018-24335-w (PMC5917029; doi:10.1038/s41598-018-24335-w)
Supplement: Supplementary file 1 — Supplementary Information [file 41598_2018_24335_MOESM1_ESM.pdf]

Yasunari, T. J., et al., *Sci. Rep.*, http://dx.doi.org/10.1038/s41598-018-24335-w (2018).

Supplementary Information

Extreme air pollution events in Hokkaido, Japan, traced back to early snowmelt and large-scale wildfires over East Eurasia: Case studies

Teppei J. Yasunari[1,2*], **Kyu-Myong Kim**[3], **Arlindo M. da Silva**[3],

Masamitsu Hayasaki[4], **Masayuki Akiyama**[5], & **Naoto Murao**[1]

[1]Faculty of Engineering, Hokkaido University, Kita-13 Nishi-8, Kita-ku, Sapporo, 060-8628 Japan

[2]Arctic Research Center, Hokkaido University, Kita-21 Nishi-11, Kita-ku, Sapporo, 001-0021 Japan

[3]NASA Goddard Space Flight Center, 8800 Greenbelt Rd., Greenbelt, MD, 20771 USA

[4]Japan Automobile Research Institute, 2530 Karima, Tsukuba, 305-0822 Japan

[5]Institute of Environmental Sciences, Hokkaido Research Organization, Kita-19 Nishi-12, Kita-ku, Sapporo, 060-0819 Japan

*Correspondence: Teppei J. Yasunari, t.j.yasunari@eng.hokudai.ac.jp

Yasunari, T. J., et al., *Sci. Rep.*, http://dx.doi.org/10.1038/s41598-018-24335-w (2018).

References

53. Akiyama, M., Otsuka, H., Akutagawa, T. & Suzuki, H. High-concentration event of $PM_{2.5}$ in Hokkaido (translated from the Japanese title). *Proceedings of the 21st Hokkaido and Tohoku Branch Meeting of Japan Society for Atmospheric Environment*, Abstract No. 15, 2 pp (2014).
54. Bosilovich, M. G., et al. MERRA-2: Initial Evaluation of the Climate. *NASA/TM–2015–104606*, 43, 139 pp (2015). (available at: https://gmao.gsfc.nasa.gov/pubs/docs/Bosilovich803.pdf)
55. Randles, C. A., et al. The MERRA-2 Aerosol Assimilation. *NASA Technical Report Series on Global Modeling and Data Assimilation*, NASA/TM-2016-104606, 45, 143 pp (2016). (available at: https://gmao.gsfc.nasa.gov/pubs/docs/Randles887.pdf)
56. Randles, C. A., *et al.* The MERRA-2 Aerosol Reanalysis, 1980 onward. Part I: System description and data assimilation evaluation., *J. Clim.*, 30, 6823-6850, doi:10.1175/JCLI-D-16-0609.1 (2017).
57. Rienecker, M. M., et al. The GEOS-5 Data Assimilation System - Documentation of Versions 5.0.1, 5.1.0, and 5.2.0. *Technical Report Series on Global Modeling and Data Assimilation*, 27, NASA/TM–2008–104606, 118 pp. (2008). (available at: http://gmao.gsfc.nasa.gov/pubs/docs/Rienecker369.pdf).
58. Colarco, P., da Silva, A. Chin, M. & Diehl, T. Online simulations of global aerosol distributions in the NASA GEOS-4 model and comparisons to satellite and ground-based aerosol optical depth. *J. Geophys. Res.*, 115, D14207, doi:10.1029/2009JD012820 (2010).

Yasunari, T. J., et al., *Sci. Rep.*, http://dx.doi.org/10.1038/s41598-018-24335-w (2018).

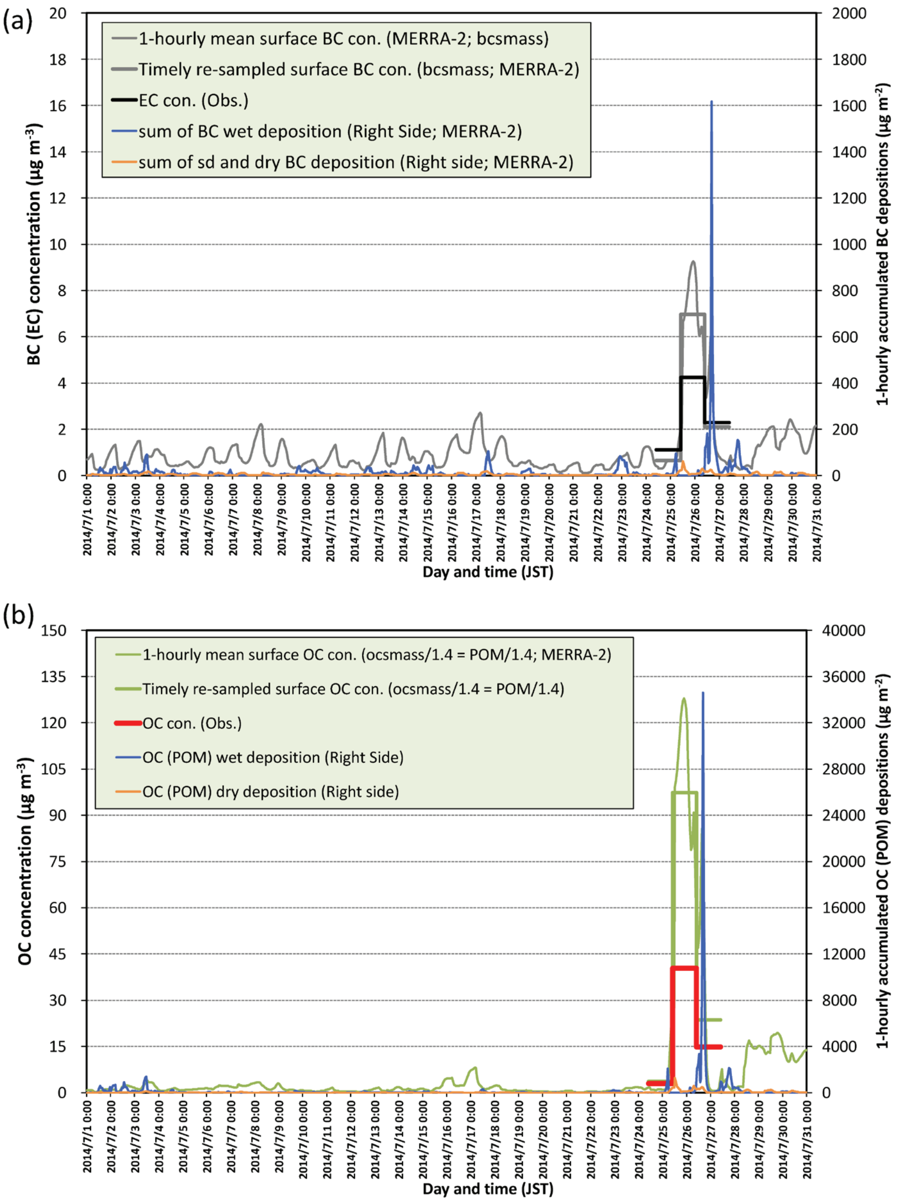

Fig. S1. Comparisons on BC and OC between MERRA-2 and observations in Sapporo in July 2014. (a) The observed Elemental Carbon (EC) (ref. 53), 1-hourly mean surface BC mass concentration (MERRA-2) and its timely re-sampled one for the time period of the observed EC (averaged during each observed interval), and BC (both hydrophobic and hydrophilic ones) wet (i.e., combined large-scale and convective scavenging) and dry depositions (i.e., combined sedimentation and dry deposition components) (MERRA-2). (b) Similar to (a) but for the observed OC (ref. 53). In GEOS-5 (i.e., MERRA-2 (ref. 54–56) was produced with GEOS-5 (ref. 57)), OC is actually Particulate Organic Matter (POM = 1.4 x OC) (ref. 58). Therefore, we divided the OC mass concentration by 1.4 to compare with the observed OC (ref. 53).

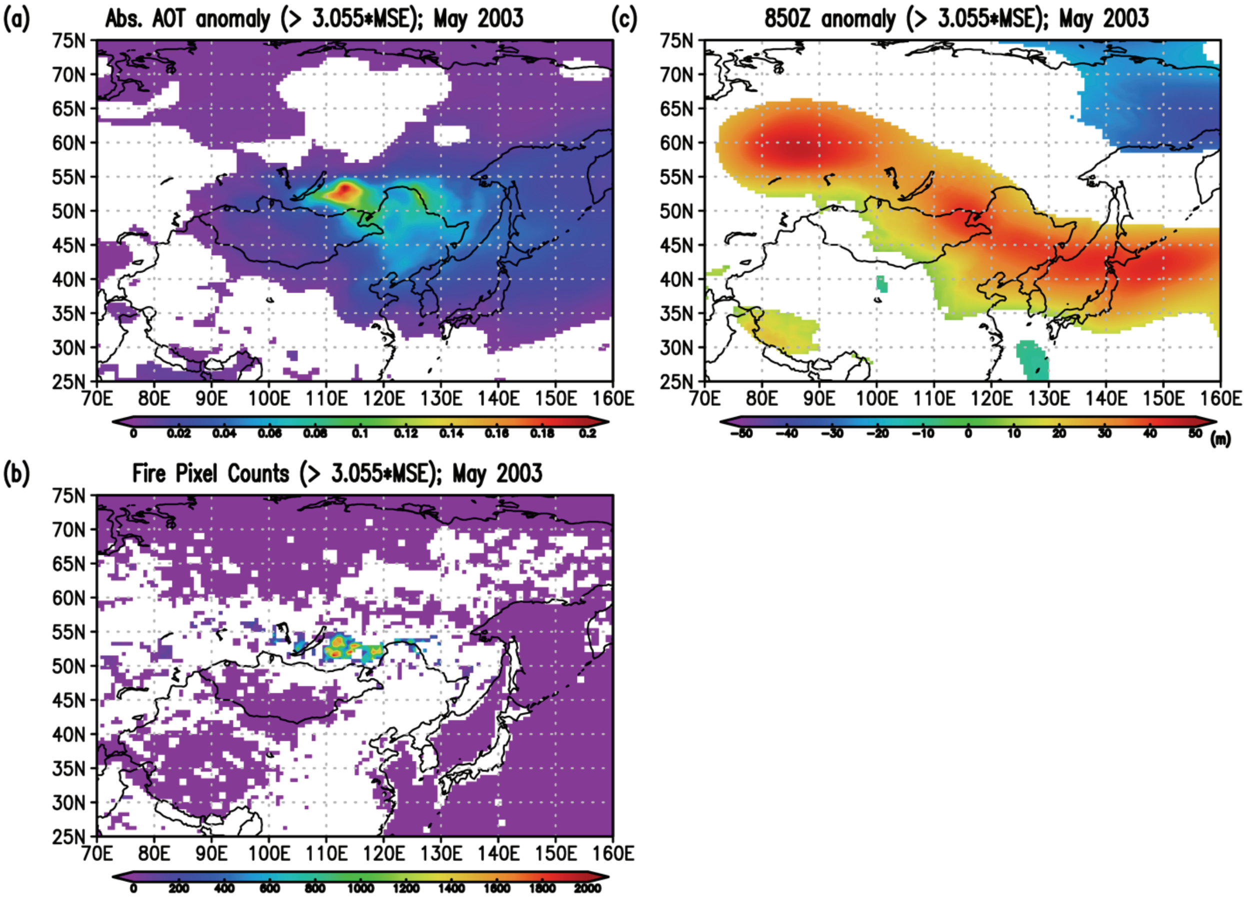

Fig. S2. The areas of the anomaly data for each component (absorbing AOT at 550 nm, fire pixel counts, and geopotential height at 850 hPa) of Fig. 3a for the wildfire case in May 2003 where the absolute values of the anomaly data were greater than 3.055*MSE (i.e., the absolute values were greater than 99% t-based confidence intervals of the climatology data). Fig. S2 was produced with OpenGrADS (http://opengrads.org/; Version 2.1.0.oga.1), which is a sub-project of the main software, Grid Analysis and Display System (GrADS; http://cola.gmu.edu/grads/).

Yasunari, T. J., et al., *Sci. Rep.*, http://dx.doi.org/10.1038/s41598-018-24335-w (2018).

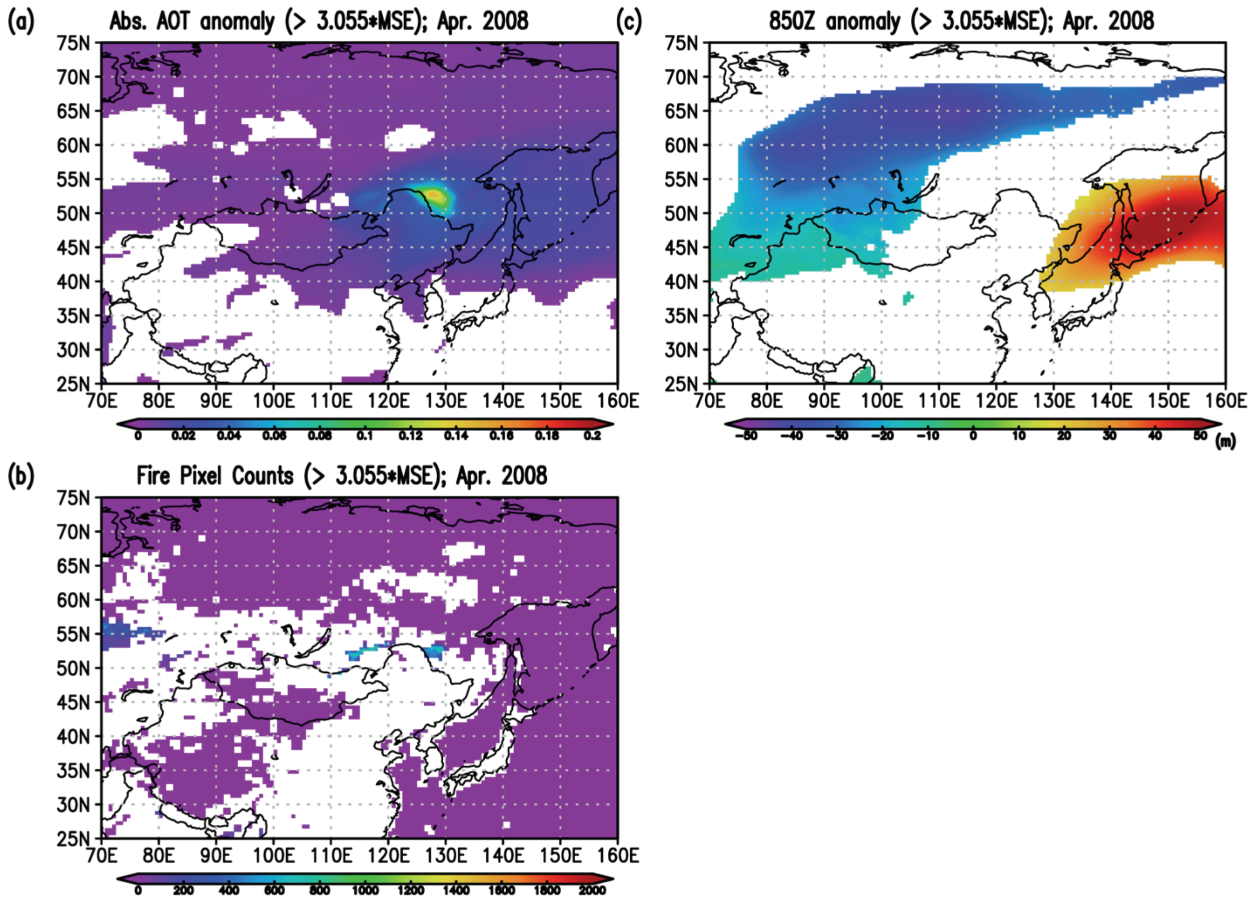

Fig. S3. Same as Fig. S2 but for Fig. 4 for the wildfire case in April 2008. Fig. S3 was also produced with OpenGrADS (http://opengrads.org/; Version 2.1.0.oga.1), which is a sub-project of the main software, GrADS (http://cola.gmu.edu/grads/).

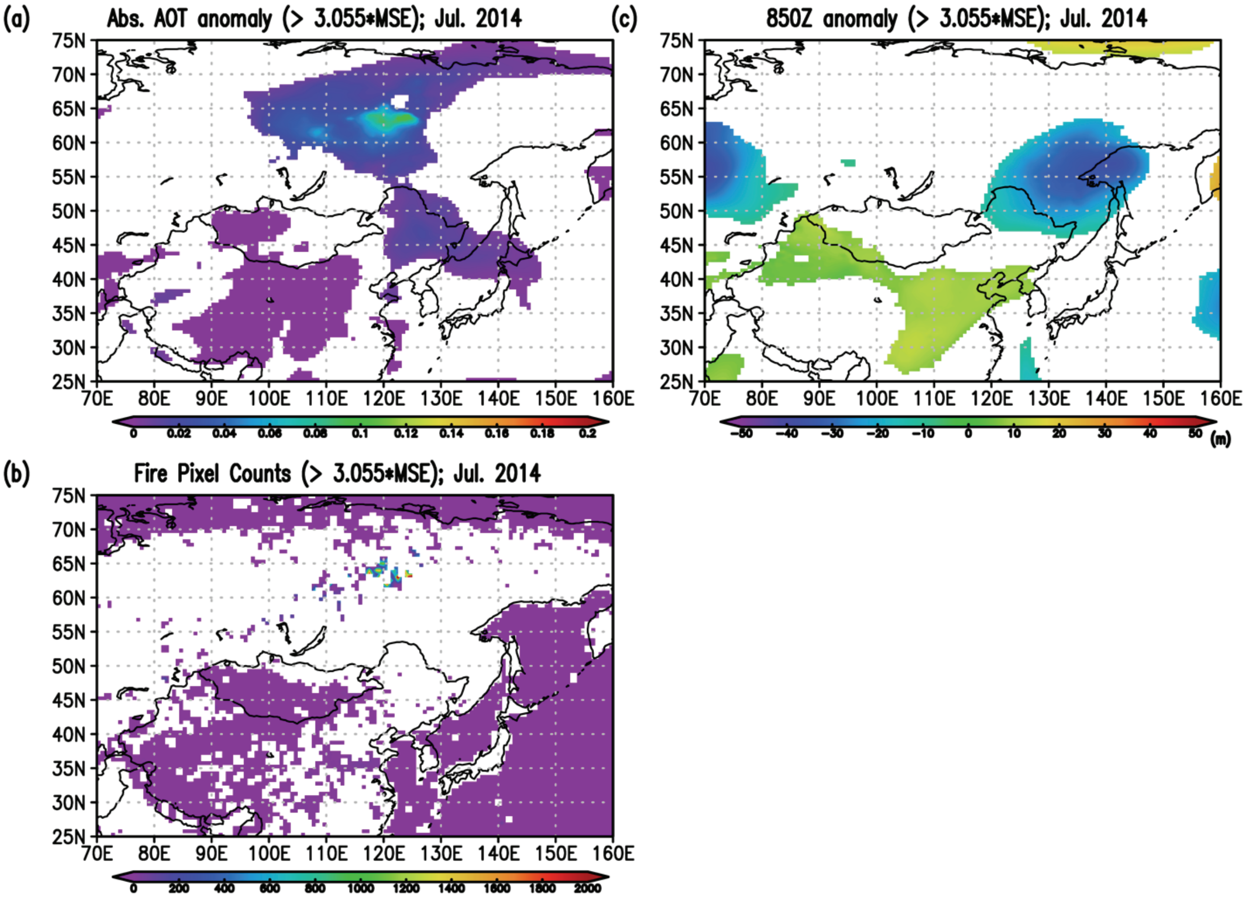

Fig. S4. Same as Fig. S2 but for Fig. 5 for the wildfire case in July 2014. Fig. S4 was also produced with OpenGrADS (http://opengrads.org/; Version 2.1.0.oga.1), which is a sub-project of the main software, GrADS (http://cola.gmu.edu/grads/).
